# Gut Microbial Composition of Pacific Salmonids Differs across Oregon River Basins and Hatchery Ancestry

**DOI:** 10.3390/microorganisms10050933

**Published:** 2022-04-29

**Authors:** Nicole S. Kirchoff, Trevan Cornwell, Staci Stein, Shaun Clements, Thomas J. Sharpton

**Affiliations:** 1 Department of Microbiology, Oregon State University, Corvallis, OR 97331, USA; thomas.sharpton@oregonstate.edu; 2 Oregon Department of Fish and Wildlife, Corvallis, OR 97330, USA; trevan.cornwell@oregonstate.edu (T.C.); staci.stein@oregonstate.edu (S.S.); shaun.clements@oregonstate.edu (S.C.); 3 Department of Statistics, Oregon State University, Corvallis, OR 97331, USA

**Keywords:** steelhead trout, gut microbiome, hatcheries, aquaculture

## Abstract

The gut microbiome may represent a relatively untapped resource in the effort to manage and conserve threatened or endangered fish populations, including wild and hatchery-reared Pacific salmonids. To clarify this potential, we defined how steelhead trout gut microbiome composition varies across watersheds and as a function of ancestry. First, we measured this variation across watersheds using wild steelhead trout sampled from nine locations spanning three river basins. While gut microbial composition differs across basins, there exist bacterial clades that are ubiquitous across all populations. Correlating the phylogenetic composition of clades with geographic distance reveals 395 clades of bacteria whose ecological distribution implicates their co-diversification with steelheads. Second, we quantified how microbiome composition varies between first generation hatchery-reared steelhead and traditional hatchery-reared steelhead. Despite being subject to the same hatchery management strategies, fish bred from wild parents carry distinct microbiomes from those bred from hatchery broodstock, implicating the role of genotype on microbiome composition. Finally, we integrated all data from both studies to reveal two distinct, yet robust clusters of community composition. Collectively, our study documents for the first time how the steelhead gut microbiome varies by geography or broodstock and uncovers microbial taxa that may indicate the watershed or hatchery from which an individual was sourced.

## 1. Introduction

Steelhead trout (*Oncorhynchus mykiss)* is an economically, culturally, and ecologically important fish. However, climate change, overfishing, and habitat destruction threaten and endanger steelhead populations. Even efforts to preserve access to steelhead through the hatchery production of fish are met with rising challenges, as fewer hatchery-reared adults return to spawn compared to their wild counterparts. Simply put, the management and conservation of Pacific salmonids faces grave challenges and may benefit from new tools that aid outcomes.

The gut microbiome is an increasingly considered but relatively untapped resource in the management and conservation of wildlife, including fisheries. Ample evidence shows that anthropogenic-caused land-use changes, climate change, environmental contamination, as well as captivity disrupts gut microbial communities [1]. This disruption is known to involve the elimination or reduction of microorganisms that are important to host health and fitness. For example, red colobus monkeys living in fragmented forests have fewer bacteria that can degrade tannins, a toxic xenobiotic present in many of their food sources [2]. The loss of these bacteria may impact their ability to digest their preferred diet and thus impact their survival. The augmentation or supplementation of microbes important to host survival and health may mitigate anthropogenic disturbances and aid conservation efforts. Therefore, learning more about the gut microbiome of vulnerable animals will embolden potential microbial related mitigation efforts with the mission of aiding threatened hosts and their microbial consortia. Knowledge of the steelhead gut microbiome is critical if we wish to use gut microbial manipulation to improve the conservation efforts related to these fish.

Despite the importance of the fish gut microbiome to their host, little is known about the steelhead gut microbiome, especially with respect to the diversity of the microbiome across distinct watersheds, wild populations, and hatchery broodstocks [3]. This paucity of knowledge challenges efforts to link the gut microbiome to management and conservation practices. Previous studies have focused on characterizing the non-anadromous member of the *O. mykiss* species, rainbow trout [4,5,6]. Additionally, previous rainbow trout gut microbiome studies have mostly been conducted in laboratory or aquaculture facilities and not in wild or hatchery populations. Thus, we were interested in characterizing the wild and hatchery steelhead gut microbiome as well as determining how the gut microbiome varies across river systems, thus informing conservation efforts regarding the necessity of location-based gut microbial interventions.

In order to characterize the steelhead gut microbiome and evaluate the gut microbial composition based on location and broodstock ancestry we conducted two studies. The first study investigated the differences between the gut microbiome of steelhead from several different locations in western Oregon. The second study investigated differences in the gut microbiome between traditional hatchery broodstock and hatchery steelhead with wild parents. We found that the steelhead gut microbiome presents geographical effects and varies based on a wild broodstock or hatchery broodstock host background, which suggests that host genotype contributes to gut microbial differences. Additionally, we reveal bacterial clades that demonstrate a phylogenetic composition in the steelhead gut that is associated with geography and that the steelhead gut microbiome has two predominant microbiome types.

## 2. Materials and Methods

### 2.1. Sample Locations

**Figure 1:**
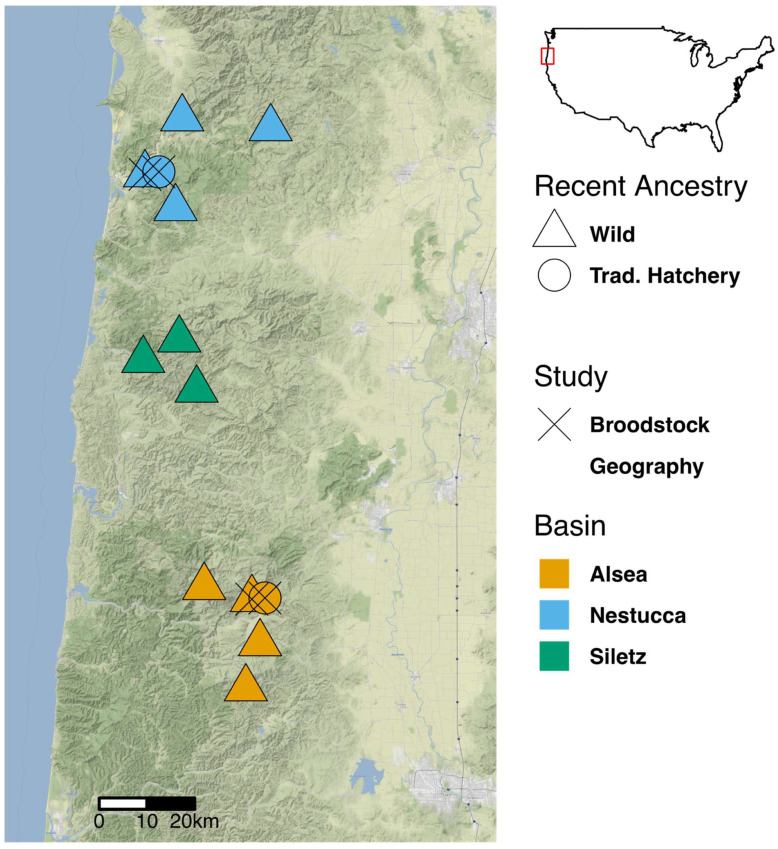
A map displaying sampling locations of wild steelhead intestinal samples. This is an image of the western Oregon coast between Portland, OR and Eugene, OR. Point shape, color, and inclusion of an “X” indicate recent ancestry, study origin, and river basin origin, respectively. The Oregon coast is located at the position of the red box on the border of the United States. Nestucca River basin samples are from the north sites in blue, Siletz River basin samples from the middle sites in green, and Alsea River basin samples from the southern sites in orange.

For the comparison of fish across river basins, we sampled ten wild-born, juvenile steelhead from each of nine freshwater systems within three Oregon river basins (Figure 1). We sampled Gravel Creek, Sunshine Creek, and Cedar Creek in the Siletz Basin; Fall Creek, Tobe Creek, and East Fork Lobster Creek in the Alsea Basin; and Alder Creek, East Fork Beaver Creek, and Elk Creek in the Nestucca River Basin.

For the comparison of wild broodstock versus traditional hatchery broodstock fish, we collected traditional juvenile steelhead from two different hatcheries as well as corresponding first hatchery generation juvenile steelhead. Specifically, thirty wild broodstock juvenile fish and thirty hatchery broodstock fish were sampled from Cedar Creek Hatchery in the Nestucca River basin and North Fork Alsea Hatchery in the Alsea River basin, respectively (Figure 1).

### 2.2. Sample Collection

For both studies, samples were collected from already scheduled steelhead sacrifices. Fish were collected with backpack electroshockers from several Oregon river basins between October 2016 and March 2017 in accordance with Oregon Department of Fish and Wildlife permits. Fish were sacrificed with a buffered tricaine methanesulfonate (i.e., MS-222) overdose, weighed, cut from anal vent to gills, and gut digesta from stomach to intestines were squeezed into 50 mL conical tubes. To preserve the DNA content, intestinal samples were first placed on ice in the field and then placed into a −20 °C freezer within four hours of sampling. Within 24 h of sampling, samples were finally moved into an −80 °C freezer.

### 2.3. Microbiome Profiling and Analyses

DNA extraction was conducted using the Qiagen DNeasy PowerSoil kit (QIAGEN, Germantown, MD, USA) with an addition of a 10 min incubation step at 65 °C, as explained previously [7]. The 16S V4 rRNA gene was amplified using Caporaso (515F/806R) primers according to previous protocols [8,9]. DNA was then quantified using a Qubit dsDNA HS kit (Thermo Fisher, Waltham, MA, USA), then pooled and cleaned with the QIAquick PCR Purification Kit (QIAGEN). Amplicons were sequenced at the Center for Quantitative Life Sciences at Oregon State University with an Illumina MiSeq (v3 chemistry) generating 300 bp paired end reads. Sequences were generated for each study on distinct flow cells.

### 2.4. Bioinformatics and Statistical Analyses

We generated an amplicon sequence variant (ASV) table by running FASTQ sequence files through the DADA2 (v 1.9.0) pipeline [10]. Separately for each study, forward reads were truncated at 240 base pairs, chimeras were removed, and bacterial taxonomy was assigned with the SILVA rRNA database (release 128) and the Ribosomal Database Project’s naïve Bayesian classifier [11]. We then created a phylogenetic tree using V4 rRNA gene sequence alignments via FastTree (v 2.1.10) [12]. We used the R (v 3.6.2) phyloseq package (v 1.3) to rarify sequence abundances for each sample within a study [13,14]. Pairwise Bray–Curtis dissimilarities for each gut microbial sample were calculated to compare abundance-weighted bacterial community compositions across sample location, steelhead weight, and management strategy using the vegan package (2.5–6) [15]. Monophyletic bacterial clades within taxonomic phylotypes were identified with the ClaaTU algorithm [16].

The non-metric multi-dimensional scaling (NMDS) plot was generated in R also using the vegan package to visualize the similarity of compositional abundance with a method that is robust to data sparsity [15]. Beta dispersion was calculated and compared with a Tukey HSD test using the vegan and stats packages, respectively. The coin package (v1.3-1) was used to conduct Kruskal–Wallis tests comparing bacterial cladal abundances across early life history categories and geographic location [17]. Multiple test correction was performed with the p.adjust() function in the stats package (v 3.6.2) with a false discovery rate cut-off of 0.05 [13]. Weighted pairwise UniFrac values were also calculated with vegan to determine the phylogenetic distance between bacterial clades present in the steelhead gut microbiome [15]. Additionally, we computed the straight line geographic distances between steelhead sample sites using the geosphere package (v 1.5-10) [18]. Hierarchical clustering (median, ward-d2) and dendrogram visualization was conducted using the stats package [13].

### 2.5. Combination of Both Studies

Data from both the geography and hatchery broodstock vs. wild broodstock studies were pooled and bioinformatically and statistically analyzed together. The combined FASTQ files were re-processed through DADA2 quality filtering, and forward reads were cut at 240 base pairs [10]. The phyloseq package was used to normalize the library size and randomly subsample (i.e., rarefy) to a maximum of 1576 reads for each sample (median reads per sample = 11,919), and 16S classification was conducted with the SILVA rRNA gene database. Phylogenetic tree inferences were conducted in FastTree, as in the two studies above [12]. Partitioning around medoids (PAM) cluster analysis was performed in R with the cluster package (v 2.1.0) [19].

## 3. Results

### 3.1. Wild Juvenile Steelhead Trout Gut Microbial Communities Are Structured by Geography and Host Fitness

**Figure 2:**
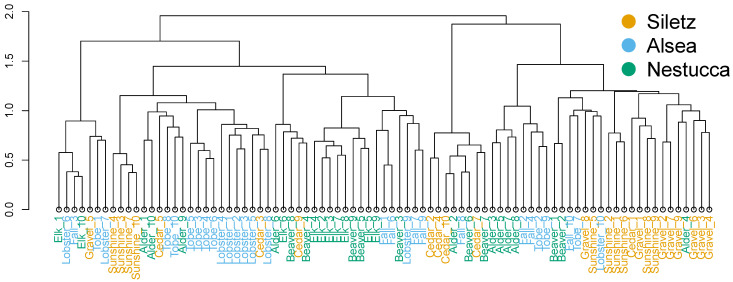
Steelhead gut microbiome samples from three different river basins in Oregon roughly group together. Dendrogram showing hierarchal clustering (Ward’s method with Ward’s clustering criterion) comparing Bray–Curtis dissimilarities between samples. Samples are colored by river basin origin. Samples do not neatly separate into three groups, but the samples tend to cluster into smaller groups with like colors. The differences between steelhead gut microbial composition are confirmed statistically (PERMANOVA, R^2^ = 0.05, *p* < 0.01).

To determine if the composition of the steelhead gut microbiome associates with steelhead geography, we rarefied to 13,635 bacterial reads and evaluated the beta diversity of the gut microbiome across locations (Supplementary Table S1). The bacterial community composition of the steelhead gut is significantly different across Oregon river basins, though the effect sizes are weak (PERMANOVA, Bray–Curtis, R^2^ = 0.06, *p* = 0.001) (Figure 2). This associative pattern is retained when comparing the beta diversity of individual sample sites, and moreover, the model improvably fits the data (PERMANOVA, Bray–Curtis, R^2^ = 0.19, *p* < 0.05). These results indicate that a steelhead’s gut microbiome is related to their geographic location, but the steelhead gut microbiome has a stronger association with the exact river or stream the fish inhabited.

**Figure 3:**
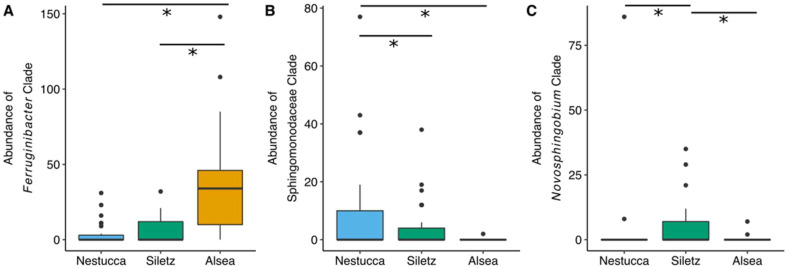
Examples of bacterial clades that are more abundant in each of the three river basins sampled in the geography study. Boxplots visualizing the abundance of steelhead gut microbiome bacterial clades across three western Oregon river basins. Asterisk (*) indicates a statistically significant result using Kruskal-Wallis tests and false discovery rate multiple test correction. (**A**) shows one clade’s abundances from the genus *Ferruginibacter*, (**B**) from the family Sphingomonadcaeae, and (**C**) from the genus *Novosphingobium*.

To discern which taxa may drive these river basin-specific patterns in community composition, we leveraged a phylogenetic approach that aggregates observed counts of ASVs among lineages that constitute monophyletic clades and applied Kruskal–Wallis tests to focus on clades whose aggregated abundances differ between river basins. In so doing, we resolved 21 bacterial clades that stratify the Alsea River basin from the Siletz and Nestucca basins. For example, a *Ferruginibacter* clade is more abundant in Alsea than in the Siletz and Nestucca basins (Figure 3A). We also found 36 clades whose abundances in the Nestucca basin differ from those in the Siletz and Alsea basins, including a clade of Sphingomonadaceae that is more abundant in the Nestucca basin (Figure 3B). Finally, we discovered four bacterial clades that differ in terms of abundance in the Siletz basin compared to the Nestucca and Alsea basins that includes one clade of *Novosphingobium*, two clades of *Aeromonas*, and one clade of *Flavobacterium* that are more abundant in the Siletz basin (Figure 3C).

Despite these differences across location, we also resolved several microbial clades that were common to all locations. In particular, we identified 1489 clades that are significantly more prevalent across samples than expected by chance (FDR < 0.05). Thirty-six of these conserved clades were present in every steelhead gut sample and they encompass taxa such as *Flavobacterium*, *Hyphomicrobium*, and *Singulisphaera*. Such microbes may manifest these ubiquitous distributions because they are common in the environment, apt at colonizing the salmonid gut, or specifically selected for by the host.

**Figure 4:**
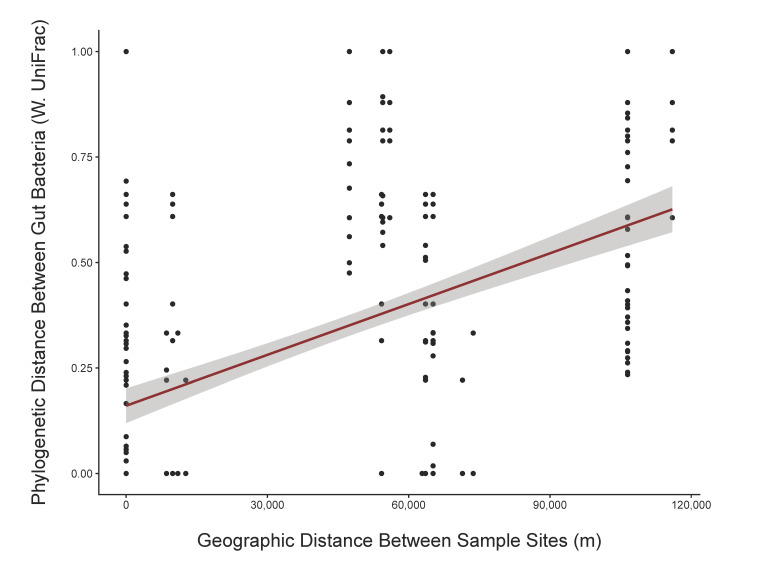
Sphingomonodaceae cladal abundance from north to south geography sampling sites shows a relationship between the phylogenetic composition of the clade and geography. Scatter plot representing a Sphingomonadaceae clade that has a significant correlation between weighted UniFrac and physical straight-line distance between coordinates of sample sites (Mantel test < 0.01). The red line represents the slope of all the data points and shows the positive relationship between geographic distance and phylogenetic distance. The shading represents the 95% confidence interval. This significant trend indicates that sampling sites that are geographically closer together tend to host bacteria with a more similar phylogenetic history. Forty other Sphingomonadaceae clades also display weighted UniFrac values that correlate with geographic distance, and Sphingomonadaceae was the taxon with the most significant clades after this analysis.

Given the pattern of variation in the salmonid gut microbiome that we observed across locations, we next sought to determine if any salmonid gut microbial clades manifest phylogenetic compositions that are statistically structured by the geography of their host, which may imply population-level co-diversification. To discern such associations, we correlated the pairwise-weighted phylogenetic beta diversity and geographic distances of steelhead gut bacterial clades. This analysis revealed 395 monophyletic clades of bacteria whose phylogenetic compositional differences across samples correlates with the geographic distance spanning sampling locations (Supplementary Table S2). The gut microbial clades that display this phylogenetic distance by geographic distance structure include members of the families Sphingomonadaceae and Rhodobacteraceae. For example, forty-one Sphingomonadaceae clades have a weighted UniFrac value that is significantly correlated with geographic distance between sample site (Mantel test < 0.01) (Figure 4). These patterns indicate that the gut bacterial phylogeny of some clades is related to the geographic location of their host. However, our analysis was based on a limited number of sampling locations and relied on a test of correlation that may be subject to relatively high type I error rates.

Some of the variation in the composition of the gut microbiome observed here could hold implications for salmonid fitness. For example, larger sized salmonids have greater reproductive success (i.e., the number of offspring that survive to maturity) compared to their smaller siblings [20]. Accordingly, a larger animal size is related to greater fitness (i.e., reproductive success) in steelhead trout. We thus determined whether the composition of the gut microbiome links to this salmonid fitness indicator through a test of association. In particular, we compared the steelhead gut bacterial structure to the weight of all fish and found that the gut microbiome is associated with steelhead weight (PERMANOVA, Bray–Curtis, R^2^ = 0.1273, *p* = 0.03).

### 3.2. Juvenile Steelhead Trout Gut Microbiome Varies as a Function of Hatchery Broodstock and Hatchery Location

Traditional hatchery broodstock are subject to several genetic bottlenecks after each successive generation compared to wild broodstock fish (i.e., F1 hatchery populations with wild parents) that only experience one generation in a hatchery facility. Despite this fact, it remains generally unknown how hatchery broodstock origins impact the composition of the gut microbiome compared to their wild broodstock counterparts. Addressing this question is critical given the fact that traditional hatchery broodstock fish are less likely to survive than their wild born counterparts for reasons we do not fully understand.

**Figure 5:**
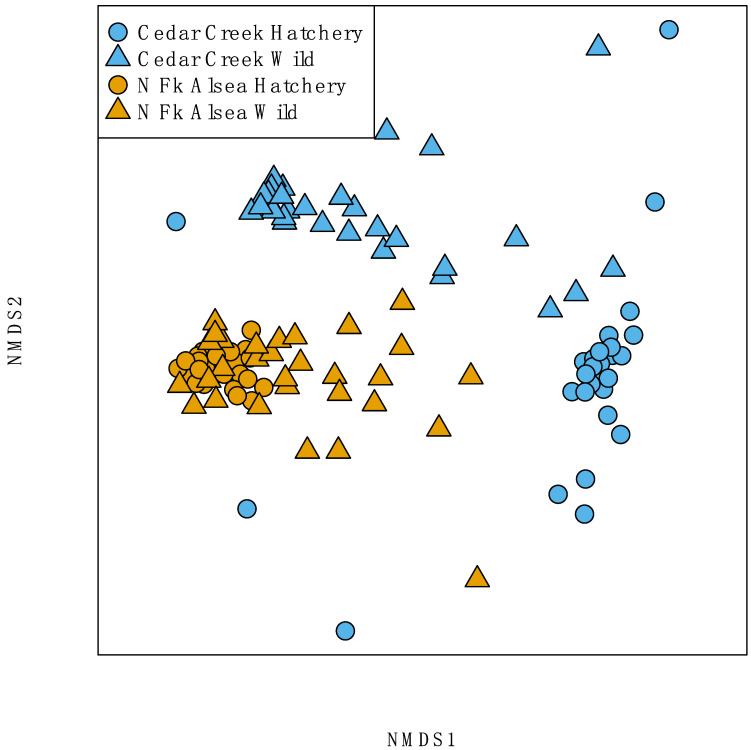
The gut microbiomes of traditional hatchery-reared steelhead differ compositionally compared to their wild broodstock counterparts. NMDS plot showcasing the differences between hatchery broodstock and wild broodstock gut microbial samples (PERMANOVA R^2^ = 0.29, *p* < 0.01) as well as differences between Cedar Creek Hatchery and North Fork Alsea Hatchery locations (PERMANOVA R^2^ = 0.07, *p* < 0.001). Stress = 0.13. Visually, there is separation between wild steelhead gut microbial composition and hatchery steelhead gut microbial composition within their respective river basins.

**Figure 6:**
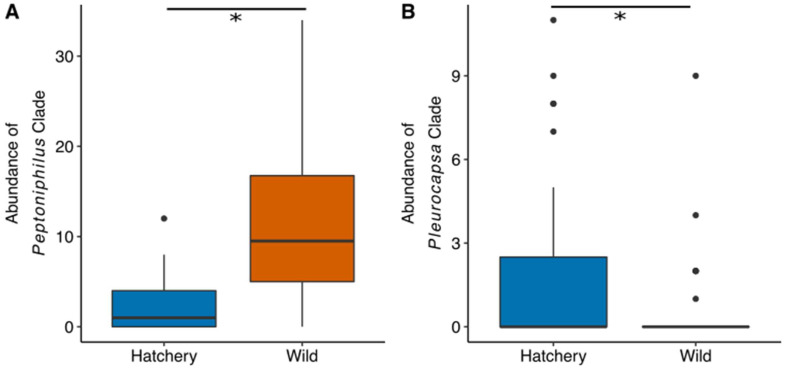
Examples of gut bacterial clades that are more abundant in either traditional hatchery or wild broodstock fish. Asterisk (*) indicates a statistically significant result using Kruskal-Wallis tests and false discovery rate multiple test correction. (**A**) This *Peptonophilus* clade example is more abundant in wild broodstock fish guts. (**B**) The *Pluerocapsa* clade example is more abundant in traditional hatchery broodstock fish.

**Figure 7:**
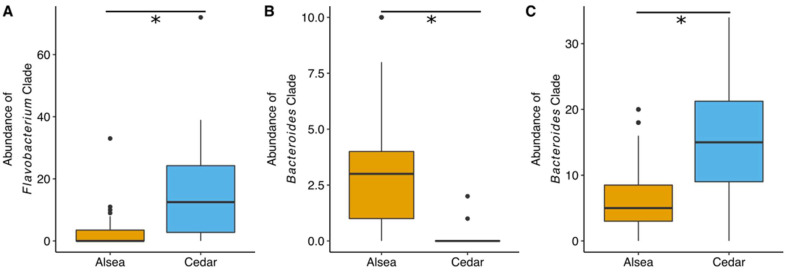
Examples of gut bacterial clades that are more abundant in all Cedar Creek Hatchery samples compared to all East Fork Alsea Hatchery samples. Asterisk (*) indicates a statistically significant result using Kruskal-Wallis tests and false discovery rate multiple test correction. (**A**) A clade from the genus *Flavobacterium* that is more abundant in Cedar Creek Hatchery fish guts. Different *Bacteriodes* clades that are (**B**) more abundant in East Fork Alsea Hatchery fish guts and (**C**) more abundant in Cedar Creek Hatchery fish guts.

After subsampling bacterial reads to 1237 reads, our analyses indicated that traditional hatchery broodstock fish carry different gut microbiome assemblages relative to their wild broodstock counterparts (PERMANVOA, Bray–Curtis, R^2^ = 0.07, *p* = 0.001) (Figure 5) (Supplementary Table S3). A total of 665 bacterial clades are differentially abundant across fish ancestry (Supplemetary Table S4). For instance, all 13 of the significant clades from the genus *Peptoniphilus* are more abundant in the gut microbiome of first-generation steelhead (Figure 6A). All four *Pleurocapsa* clades are more abundant in the guts of traditional hatchery broodstock steelhead (Figure 6B). Additionally, there appear to be hatchery-specific effects on the interindividual variation of the microbiome. For example, the NMDS plot of beta diversity shows that traditional North Fork Alsea Hatchery samples are more tightly gathered than the North Fork Alsea Hatchery wild broodstock samples. Thus, we compared the beta dispersion of the steelhead gut microbial samples and found that North Fork Alsea Hatchery wild broodstock samples are more dispersed than the traditional hatchery broodstock samples (Tukey HSD of beta dispersion <0.001). This differentiation in dispersion could contribute to the observed differences in beta diversity. Furthermore, we determined that the gut microbial structure of steelhead is also associated with their creek of origin, irrespective of their hatchery or wild broodstock status (PERMANOVA R^2^ = 0.29, *p* < 0.01), suggesting that specific aquatic environments play a role in shaping steelhead gut microbial structure. The contribution of geographic origin may also explain the overlap of North Fork Alsea Hatchery samples visible in the NMDS plot that is not seen between Cedar Creek Hatchery traditional broodstock samples and Cedar Creek Hatchery wild broodstock samples, as both North Fork Alsea Hatchery broodstocks were established using fish from the Alsea River and the Cedar Creek hatchery fish were established using fish from two different locations. There are 1664 bacterial clades with different abundances between the North Fork Alsea Hatchery and Cedar Creek Hatchery locations (Supplementary Table S5). All 29 of the clades assigned to the genera *Flavobacterium* are more abundant in the Cedar Creek Hatchery location (Figure 7A). Furthermore, most of the 33 clades assigned to the genus *Bacteroides* are also more abundant in the Cedar Creek Hatchery samples, but eight of the clades are more abundant in the North Fork Alsea Hatchery location. We visualized the abundance distributions of one of the *Flavobacterium* clades and one of the *Bacteroides* clades (Figure 7B). Collectively, our results indicate that broodstock generation and watersheds impact the assembly of the steelhead gut microbiome.

### 3.3. Combination of Both Studies

**Figure 8:**
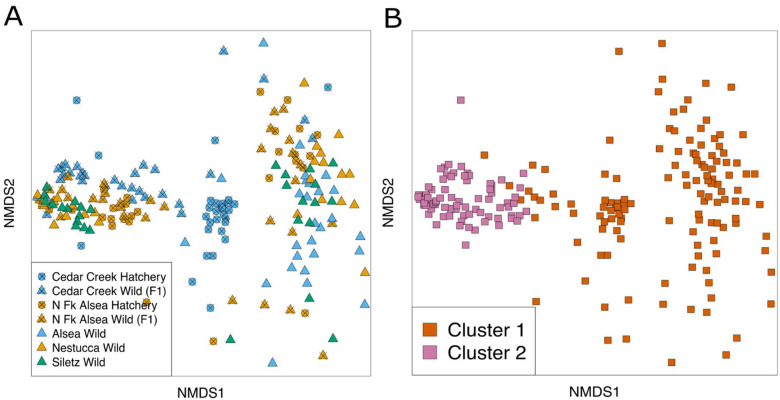
NMDS plots and PAM cluster analysis reveal two clusters that may represent two steelhead gut microbiome types. (**A**) shows the combined NMDS visualization of all wild-born and hatchery-reared steelhead gut microbiome samples. The samples aggregate into two groups separated by space in the ordination. The coloring based on river basins and the shapes based on management type indicate that neither of these variables separates out into these two clusters. (**B**) colors each microbiome sample based on the PAM cluster designation and shows that PAM cluster assignment corresponds with the two speculated clusters.

After combining all our available wild-born, wild broodstock, and hatchery broodstock gut microbiome samples, we found that the river basin, broodstock history, and weight remained associated with the beta diversity of the steelhead gut microbiome (PERMANOVAbasin, Bray–Curtis, R^2^ = 0.1223, *p* = 0.001; PERMANOVAbroodstock, Bray–Curtis, R^2^ = 0.0479, *p* = 0.0001; PERMANOVAweight, Bray–Curtis, R^2^ =0.1170, *p* = 0.0001). Additionally, the dimensions displayed in the NMDS show two all-encompassing potential clusters that we confirmed with a partitioning around medoids (PAM) cluster-based analysis (Figure 8). Despite their separation, these clusters are not explained by any of our measured variables, suggesting some other variables underlie this observed structure in the diversity of the steelhead gut microbiome.

## 4. Discussion

Pacific salmonid fisheries have the task of keeping up with consumer demands as wild and hatchery population numbers decline. Understanding how the Pacific salmonid gut microbiome varies based on broodstock ancestry or geographic location will provide insight into how gut bacteria may be manipulated to improve fish health and survival. This study defines how the steelhead trout gut microbiome varies across three river basins and as a function of their broodstock background. In particular, this study reveals geographic, geographic by phylogenetic lineage, and ancestry effects on the steelhead gut microbiome. Additionally, this study found an association between the steelhead gut microbial community and weight, which may have fitness implications for these fish. We document several bacterial clades that stratify groups with differing gut microbial diversity. Finally, a combined analysis revealed two predominant types of steelhead gut microbiome composition. This work clarifies how geographic location and broodstock affect the steelhead gut microbiome and informs our understanding of how the gut microbiome manifests in declining fish populations, which may lead to improved management practices or conservation efforts.

This study highlights the existence of geographic effects that influence the composition of the gut microbiome. These observations generally agree with prior studies of wildlife gut microbiomes in terrestrial systems [21,22], and a recent meta-analysis revealed differences in the gut microbiome of over 85 species of fish based on the five Korean water sources they were sampled from [23]. Another study, though, found that the wild gut microbiome of Atlantic salmon did not associate with geographic location [24]. However, our study was conducted with a larger sample size of Pacific salmonids, suggesting a larger effect size many be needed to reveal geographic patterns in salmonids or that differences between Atlantic and Pacific salmonids—such as differences in physiology, ecology, or geography—may account for these distinct results.

Importantly, cryptic variation in host physiology or genetic background may shape the gut microbial composition in this study observing wild-born gut microbes. Salmonids show evidence of subpopulations and genetic differences even within the same river system, and genetic differences have been seen in trout with spawning habitats as low as 2 km apart [25,26]. Given that the host genotype plays a role in shaping gut microbial composition in other fish hosts, the differences seen in the gut microbiome across geographic locations may be related to the accompanying differences in host genetics [27]. However, the steelhead genetics of the wild samples were not explicitly documented in this study, and future work should attempt to correlate gut microbial members with genetic differences in wild steelhead trout.

We uncovered that specific bacterial clades were more abundant in one of the three river basins, which supports the hypothesis that gut microbes may be useful for assessing salmonid biogeography. For example, a clade belonging to the genera *Ferruginibacter* was more abundant in the Alsea Basin, a Sphingomondaceae clade was more abundant in the Nestucca Basin, and *Novosphingobium* was more abundant in the Siletz Basin. In a previous study, *Novosphingobium* abundance varied by geographic location in the gut of another fish species, suggesting that members of this bacterial genera typically show geographic patterns within the fish gut microbiome [28]. While the function of *Ferruginibacter* in the fish gut is unknown, bacteria from this genus are often isolated from freshwater sediment, suggesting that these bacteria are dispersing from sediment to fish gut or from fish gut to sediment [29]. Regardless, given the cross-sectional nature of our study, it is not clear if these geographic associations are maintained over the course of a fish’s lifespan, a topic that should be explored in future work.

An additional analysis revealed several clades that display correlations between geographic distance and phylogenetic distance in the steelhead gut microbiome, suggesting that these gut bacterial members co-diversified with their hosts. Alternatively, these bacterial clades may manifest a geographic distribution in the environment and then occupy the fish host. Clades demonstrating this geographic and phylogenetic correlation include clades from the bacterial families Rhodobacteraceae and Sphingomonodaceae. Rhodobacteraceae may play a role in fish health, as this family was previously found to be more abundant in the guts of healthy shrimp compared to diseased shrimp [30], but future studies should explicitly test its role in steelhead health. Also, bacterial clades from Sphingomonodaceae produce sphingolipids, which are organic compounds that can modulate *O. mykiss* mucosal homeostasis and B cell abundance [31]. Although we did not sample mucosal-associated bacteria, mucosal membranes and digesta share some microbial members and microbes in the digesta and lumen can still produce compounds that affect host immune responses [32]. Additionally, Sphingomonadaceae possess sphingolipids in their cell membranes that improve chances of successful colonization and survival in the gut, which can be advantageous for both commensal and pathogenic organisms [33,34]. This speculative role of Rhodobacteraceae and Sphingomonadaceae may be the reason for a potentially prolonged association between these bacterial families and steelhead that gave rise to this geographic lineage sorting.

Despite differences in gut microbial structure across basins, we discovered bacterial clades that are prevalent in all steelhead guts of our first study. Bacterial clades from the genera *Flavobacterium*, *Hyphomicrobium*, and *Singulisphaera* represent such core taxa. The ubiquitous presence of these bacteria suggest steelhead physiology selects for these specific clades, as they may have critical functions within the steelhead gut, or that these bacterial clades are also commonly found in the surrounding aquatic meta-communities. The function of *Hyphomicrobium* and *Singulishpaera* in the gut are unknown, but they have been found in aquatic systems as well as other fish guts [35,36]. Several members of the *Flavobacterium* genus are pathogenic to fish, although some *Flavobacterium* are commensal [37]. The pathogenicity of the *Flavobacterium* clades in this study is unclear but could have widespread consequences, as these clades were found in every fish of our first study. Given their ubiquitous distribution, future investigations should seek to discern the physiological impacts of these taxa.

In addition, this study clarifies the impact of hatchery broodstock on the gut microbiome. Previous work suggested that hatcheries elicit strong selective pressure on Pacific salmonids that differentiate fish reared in hatcheries for several generations from fish reared in hatcheries for one generation, who are both different from wild-born salmonids. For example, the relative fitness levels and rates of reproductive success of fish with greater hatchery ancestry are significantly lower than those of fish with wild ancestry [38]. Also, the expression of several genes from the first generation of hatchery steelhead trout (i.e., previously wild trout) are heritably altered after a single generation in a hatchery environment [39]. The differences in the diversity of traditional hatchery and first-generation hatchery gut microbiomes suggest this selective pressure is also applied to steelhead gut microbial communities. Other heavily managed animals are known to have different gut microbial communities compared to their wild counterparts, as is the case with animals in captivity [40].

The fact that the first-generation hatchery stock and traditional hatchery stock were reared in the same hatchery environment suggests that the differences in the gut microbiome between these two groups is due to differences in genetics. Genotype has previously affected gut microbial composition in fish and other hosts [27,41]. Future conservation efforts may use the identification of specific clades that stratify or are indicative of a hatchery or wild steelhead gut microbiome to identify a fish as early generation or traditional hatchery-reared. Additionally, our resolution of clades that differentiate traditional hatchery and first-generation hatchery fish microbiomes may help hatcheries develop management practices that ultimately normalize the composition of hatchery-reared microbiomes closer to their wild counterparts. This study only focused on the gut microbiome of juvenile steelhead as this is the life stage steelhead are contained in hatcheries, and more mature steelhead undergo a great deal of physiological changes in preparation to travel out to the ocean, which may induce changes in the gut microbiome.

A combined analysis using all steelhead gut microbiome samples from our two studies revealed two robust clusters that demonstrate densely populated areas in the multidimensional space of steelhead gut microbiome beta diversity. These two clusters may be evidence of two different steelhead gut microbiome types. However, an unknown covariate that we did not measure, such as the sex of the fish, may be responsible for the clustering. Future studies may find these two clusters are robust among other populations of steelhead gut microbiomes and they should focus on measuring more variables that may be causing these clusters. If these two clusters are robust, future researchers should consider that the effectiveness of microbial interventions may be different based on the steelhead microbiome type measured in future studies. Therefore, microbiome type should be another variable considered when studying the steelhead gut microbiome.

Our results indicate that the steelhead gut microbiome varies as a function of geography and broodstock ancestry. Additionally, several steelhead gut bacterial clades show geographic lineage sorting across western Oregon, and a collective analysis showed two gut microbiome types. Given the declining populations of wild salmonids and the comparatively poor fitness of successive generations of hatchery-reared supplementation stock, characterizing the gut microbial communities across these populations is critical in learning 1) how the steelhead gut microbiome plays a role in the health and fitness of these fish and 2) how we can use steelhead gut microbiota or microbial interventions to improve conservation and supplementation efforts.

## Data Availability

DNA and R code generated during this research is located on the Sharpton Lab Repository (http://files.cqls.oregonstate.edu/Sharpton_Lab/Papers/Kirchoff_Microorganisms_2022/ accessed on 22 April 2022).

## Supplementary Materials

The following supporting information can be downloaded at: https://www.mdpi.com/article/10.3390/microorganisms10050933/s1, Table S1: Number of reads before and after rarefaction for the geography study; Table S2: Table of clades that were correlated with phylogenetic distance and geographic distance. The table includes mantel test *p*-values and associated bacterial taxa identification; Table S3: Number of reads before and after rarefaction for the broodstock study; Table S4: Gut microbial clades significantly different across traditional hatchery broodstock and wild broodstock fish; Table S5: Gut microbial clades significantly different between North Fork Alsea Hatchery and Cedar Creek Hatchery.
